# Multi-faceted identities and interactions in mixed health teams

**DOI:** 10.1186/s13584-016-0092-5

**Published:** 2016-07-19

**Authors:** Daniella Arieli, Miriam J. Hirschfeld

**Affiliations:** Department of Nursing & Department of Sociology and Anthropology, Max Stern Academic College of Emek Yezreel, Emek Yezreel, Israel; Nursing Department, Haifa University, HaRav Herzog St. 22, Tel-Aviv, 62915 Israel

## Abstract

The literature in the area of the health workforce and societies in conflict encompasses a wide range of studies and potential directions. Lately, Keshet and Popper-Giveon reported on a study based on interviews with 13 Arab Israeli nurses who work in Israeli hospitals. This preliminary study describes how being an Arab nurse in Israel is experienced and perceived by those nurses. The results indicate the need for further studies on the complexity of health workers’ experiences in their changing and multi-faceted professional, cultural, gender and national identities. In order to manage health systems, in particular in divided societies that are characterized by inter-group conflicts, special attention should be given to studying the everyday processes in mixed teams.

## Background

The article by Keshet and Popper-Giveon [1] focuses on the experiences of 13 Arab nurses working in public hospitals in Israel. Based on deep interviews with them, the authors discuss the interlinking professional and ethnic identities of the interviewees. From this paper one can learn that the Arab nurses are content with their professional identity and see nursing as a good occupational opportunity for themselves, their families and communities and for the population of Arab speaking patients in general. However, the paper presents also the difficulties these nurses experience, such as negative stereotypes and suspicion towards them from some of the Jewish patients, as well as stress and tensions in the relationships between them and some of their Jewish colleagues, in particular at times when the Israeli - Palestinian conflict erupts and becomes more violent. The interviewees report coping strategies that include refraining from expressing negative emotions and a tendency to downplay the importance of the conflictual issues.

### Directions for further research

This preliminary study raises questions that call for further research. The scientific literature in the area of the health workforce and societies in conflict encompasses a wide range of studies and potential directions, from the involvement of the World Health Organization and NGOs in trying to provide emergency care and build peace [2], to conflicted identities of minority workers in different health systems and discrimination against migrant nurses [3–5] to differential care of populations in areas of ethnic strife [6]. In this commentary we shall merely highlight a few selected studies in order to suggest future research directions.

One main direction for further research, based on the Keshet and Popper-Giveon’s article, is an exploration of the complexity of health workers’ experiences in their changing and multi-faceted professional, cultural, gender and national identities. Conducting such studies requires researchers to address ambivalent and complex aspects of identities and to ask how workers’ experiences differ according to gender, profession and changing institutional and national/regional environments.

The case of Palestinian/Arab citizens of Israel who work as nurses (as well as physicians or pharmacists etc.) presents a particularly complex example of multi-faceted and ambivalent identity. Based on 108 in-depth interviews of Palestinian women who are Israeli citizens, Herzog [7] found continual ambivalence and confusion of crossing and reconstructing boundaries in encounters with the dominant society. In a paper titled “Being an Arab citizen in Israel: Characteristics, challenges and obstacles” [8], Abu-Riya describes the Arab citizens of Israel as coping with a sense of alienation which results from, among other reasons, the fact that the symbols of the state are representing the Jewish nation and from the attempts of the state to oppress the Palestinian identity which many of them identify with. Herzog and Abu-Riya’s findings raise the question of how relevant these results are for Arab citizens working in the Israeli health system; and to what extent feelings of belonging to an organization depends upon the system allowing open expression of opinions and showing complex identities.

In the case of Israel, these questions do not have an easy answer, and apparently the main strategy in the Israeli health system where mixed teams of Jews and Arabs work together is to refrain as much as possible from addressing issues that might raise tensions. The focus becomes the common professional denominator i.e. the common goal of providing good care to patients. And indeed, this strategy works, since in spite of the difficulties and the tensions, which Keshet and Popper-Giveon describe, mixed medical teams of Jews and Arabs succeed not only in functioning reasonably well, but also in managing successful work relations in conditions of violent and continuing national conflict [9]. The entire Israeli health care system relies on the work of such mixed teams and on the professional cooperation on which they are based.

Alas, our knowledge regarding the interpersonal everyday processes in mixed teams is still wanting. Research based on deep interviews, such as those reported in the paper of Keshet and Poper-Giveon add to our understanding by describing the subjective experiences of team members and giving voice to those who are seldom heard in the public space. But the dynamic interactions that close working relations consist of, and the complex situations health workers meet daily, need far more research attention.

Many questions are waiting to be addressed, as for instance: What happens in the verbal and nonverbal communication among staff members? What happens in moments of tension and conflict and how do people reconcile tensions? What is spoken and what remains unspoken? Who does the talking and with whom, and who are those who never talk? What are the measures that succeed in fostering cohesion among team members and what happens when team members feel that others do not accept them or do not accept some parts of their identity?

Furthermore, one must also ask how do patients (who also carry their own complex and multifaceted identities and identifications) deal with diverse and ethnically different health professionals, how does it affect their trust and health behaviors and how do the complex relations between team members affect the work with patients who come from various backgrounds? And perhaps more important, how does management promote, if not provide, salutogenic environments for patients, families and the health workforce?

In order to learn more about these dynamics and to provide a glance into what is “hiding behind the curtain” there is a need for researches based on ethnography. The aim of ethnographic research is to try and see life as it happens, or in other words to watch how people act in their natural environment and to bring “situated knowledge” [10] to the research field. Ethnographic research is based upon participation of the researcher in the field of study over time, on observation of the daily routines and documentation of situations occurring, and on the analysis of the meanings in the context of the various situations. Such studies require researchers who are willing to spend extensive time in health institutions and also demands that health institutions facilitate, encourage and support such studies.

Such studies might allow us to learn from the apparent success of the health care system in Israel to date. However, the positive atmosphere that by and large characterizes the Israeli health system should not be taken for granted. The continuing conflict and the escalating extremism in many parts of Israeli society warn us to not ignore the explosive potential of this situation. It seems prudent to conduct research on what is actually going on, in the hope of finding ways to pro-actively ameliorate the tensions.

Studying interactions in mixed health and nursing teams is an important topic not only in Israel, but globally. This direction for research and intervention is also rooted in the global health workforce crisis. In its World Health Report [11], the WHO addressed the gaps of available health workers and the exodus of skilled professionals from poor to rich countries in the midst of much unmet health need in poor regions. This is a complex issue with serious ethical dimensions, including fairness, social solidarity, individual choice and, last but not least, the viability of national health care systems and their potential to provide equitable health care services. While raising more specific research questions here is beyond the specific scope of this commentary, one minor, but important view is the analysis of experiences of immigrant and minority health workers.

The British NHS has been recruiting black and minority ethnic nurses from former colonies over decades. In a phenomenological study Alexis, Vydelingum and Robbins [3] found that these nurses felt discriminated against, devalued and indicated that white UK nurses had little trust in them. Yu Xu [4] documented racism and discrimination in a meta-synthesis of 14 studies of lived experiences of immigrant Asian nurses to western countries and raised questions of how not only social justice, but also work team cohesion could be achieved by honest dialogue [5]. An issue of no less importance is raised by Mohammed & Angell [12] who suggest that the increased use of teams, coupled with an increasingly diverse workforce calls for studies on how team diversity affects functioning and performance. De Dreu [13] argues against the positive effects of workplace conflict and, while conflict might be unavoidable, research on cooperative conflict management is needed to find ways to minimize the harm of conflict to individuals and the workplace.

We are fortunate in Israel that nursing attracts young people from minority groups and serves as an upward mobility conduit for members of the Arab Palestinian minority, as well as for members of other minority groups, such as Russian speaking immigrants and immigrants from Ethiopia. We shall be even more fortunate when the nursing profession will also attract more young people from the strong and affluent population sections.

Alas, work force diversity and adequate representation of the various groups are not enough. Cultivating the sense of belonging of all health workers seems essential to future sustainability of quality care. This seems to be of particular importance in divided societies and, even more so, in societies living in conflict. In previous studies we have described interventions that were intended to provide nursing students with efficient tools to cope with the inter-group and inter- cultural encounter that they will face when they graduate and start working in the Israeli public health system [14, 15]. These studies have pointed to the dilemmas and challenges faced when trying to achieve cultural safety: a state of mutual respect where both professionals as well as patients from diverse backgrounds feel safe to express their identities [16, 17].

## Conclusions

The concept of Health as a Bridge for Peace was embraced by international organizations such as WHO already in the 1990’s (WHO HAC) and training in various countries with societies in conflict (e.g. the Caucasus/Russia, Sri Lanka, Indonesia and the former Yugoslavia) was also facilitated. These efforts remain, however, minimally operationalized and funded. Much could be learned from well-designed intervention studies and a better understanding of how to manage and minimize the enormous cost of conflict to health and wellbeing.

## Abbreviations

NGO, Non Governmental Organizations; WHO, World Health Organization
